# Supplementary material for: Anti-Melanogenic Effects of Lilium lancifolium Root Extract via Downregulation of PKA/CREB and MAPK/CREB Signaling Pathways in B16F10 Cells
Source: Plants (Basel). 2023 Oct 24;12(21):3666. doi: 10.3390/plants12213666 (PMC10648933; doi:10.3390/plants12213666)

Figure S1. Effects of *Lilium lancifolium* (*L. lancifolium*) root extract on melanogenesis-related genes in B16F10 cells. **(A)** B16F10 cells were treated with the indicated concentrations of LRE and α-MSH for 24 h. mRNA levels of tyrosinase-related genes (*tyrosinase*, *Tyrp1*, and *Tyrp2*) were detected via qRT-PCR, with *Gapdh* serving as a loading control. **(B)** B16F10 cells were seeded in 60-mm dishes (2 × 10^5 cells) and incubated for 24 h. The cells were treated with the indicated concentrations of LRE and α-MSH for 24 h. The mRNA levels of *Mitf* were detected via qRT-PCR, with *Gapdh* serving as a loading control. The results are presented as the mean ± SD of three independent experiments and were analyzed using a one-way analysis of variance followed by Tukey's test. qRT-PCR, quantitative real-time polymerase chain reaction; mRNA, messenger RNA, Mitf, microphthalmia-associated transcription factor. $^{###,***}p <$ 0.001, $^{**}p < 0.01$, $^{*}p < 0.05$.

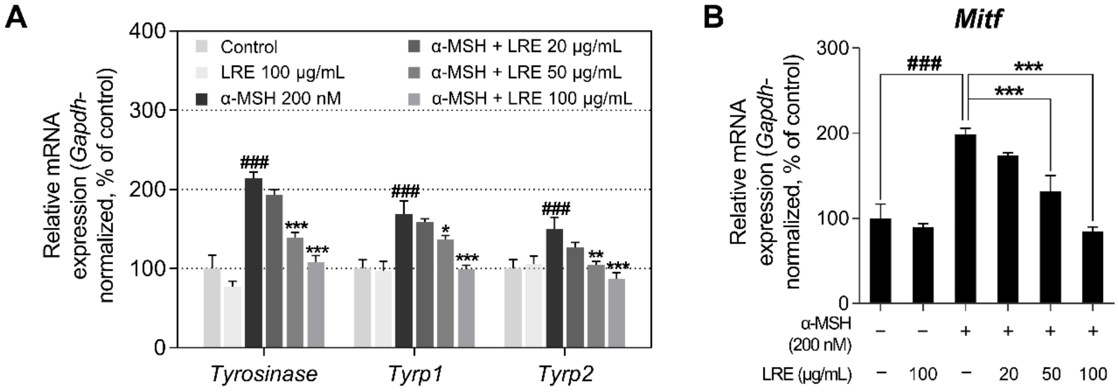

Supplement: Supplementary file 1 [file plants-12-03666-s001.zip › plants-2646316-supplementary.pdf]
